# Beneficial Effects on Exercise Capacity Associated with a Combination of Lactoferrin, Lysozyme, Lactobacillus, Resveratrol, Vitamins, and Oligoelements in Patients with Post-COVID-19 Syndrome: A Single-Center Retrospective Study

**DOI:** 10.3390/jcm13154444

**Published:** 2024-07-29

**Authors:** Alberto Maria Marra, Federica Giardino, Andrea Anniballo, Simona Ferazzoli, Andrea Salzano, Michele Arcopinto, Roberta D’Assante, Andrea De Mare, Giorgia Esposito, Lavinia Saldamarco, Sara Rurgo, Giovanni Sarnelli, Antonio Cittadini

**Affiliations:** 1 Division of Internal Medicine and Metabolism and Rehabilitation, Department of Translational Medical Sciences, University of Naples Federico II, 80131 Naples, Italy; albertomaria.marra@unina.it (A.M.M.); federica.giardino@unina.it (F.G.); aanniballo99@gmail.com (A.A.); s.ferazzoli@studenti.unina.it (S.F.); andre.salzano@gmail.com (A.S.); michele.arcopinto83@gmail.com (M.A.); andrea.demare@gmail.com (A.D.M.); gio.esposito225@hotmail.it (G.E.); 2 Interdisciplinary Research Centre on Biomaterials (CRIB), University of Naples Federico II, 80131 Naples, Italy; giovanni.sarnelli@unina.it; 3 Interdepartmental Center for Gender Medicine Research—GENESIS, University of Naples Federico II, 80131 Naples, Italy; roberta.dassante@unina.it; 4 Department of Emergency Medicine, “Betania” Hospital, 80147 Naples, Italy; lavinia.sal77@gmail.com; 5 Department of Clinical Medicine and Surgery, University of Naples Federico II, 80131 Naples, Italy; sararurgo91@hotmail.it

**Keywords:** post-COVID-19 syndrome, PIRV-F20^®^, natural supplement, exercise impairment

## Abstract

**Background/Objectives**: Although long-term COVID-19 symptoms are common, little is known about the management of post-COVID-19 condition. The aim of the current report is to evaluate the effects of a combination of lactoferrin, lysozyme, lactobacillus, resveratrol, vitamins, and oligoelements (PIRV-F20^®^) on the exercise capacity of post-COVID-19 patients. **Methods**: A retrospective analysis of consecutive patients referred to a specific outpatient clinic dedicated to post-COVID-19 condition from April 2022 to April 2023 was conducted. Subjects of both sexes, aged ≥18 years, with previous COVID-19 in the preceding 12 months, persistent symptoms consistent with post-COVID syndrome, and initial exercise impairment were included. Exclusion criteria were as follows: active cancer, end-stage conditions, severe musculoskeletal conditions, or patients with a history of limited functional capacity, pregnancy, or breastfeeding. Patients who reported having taken PIRV-F20^®^ for at least 6 weeks were compared to patients who refused this treatment. Six-minute walking distance was the primary endpoint. **Results**: Forty-four patients (56.8% women, aged 49.1 ± 18.1 years) were included in the study. The group of patients who reported having taken PIRV-F20^®^ exhibited a significant improvement of 6MWD (median: +40 m; IQR: 10–65 m, p vs. baseline: 0.02), which was significantly superior (p: 0.01) when compared to the controls (median: +10 m; IQR: −5–30 m). No differences were found with regard to muscular strength, echocardiographic parameters, and perception of symptoms. **Conclusions**: Post-COVID-19 individuals who reported having taken PIRV-F20^®^ for at least six weeks showed a significant improvement in exercise capacity. This finding should be confirmed in larger, prospective, randomized controlled trials.

## 1. Introduction

The 2020 global pandemic of coronavirus disease 19 (COVID-19), due to the wide spread of the severe acute respiratory syndrome coronavirus 2 (SARS-CoV-2), was a dramatic stress test for healthcare systems worldwide. The most common clinical presentation of COVID-19 involves the respiratory system, spreading from a mild upper respiratory tract infection with fever, dry cough, and sore throat up to interstitial pneumonia, leading to Acute Respiratory Distress Syndrome (ARDS) and respiratory failure [1,2]. However, it is largely acknowledged that COVID-19 may involve different organs since the cardiovascular system, gastrointestinal tract, kidney, thyroid gland, and testis are related to the diverse symptoms associated with this infection [3,4]. People who develop non-lethal COVID-19 usually completely recover within 4 weeks from acute infection due to SARS-CoV-2. However, it has been estimated that at least 10–30% of non-hospitalized patients, up to 70% of hospitalized ones, and 10–12% of vaccinated subjects still experience the persistence of some of the initial symptoms after this period, leading to a deterioration of quality of life [5,6,7]. In a longitudinal prospective cohort study of non-hospitalized COVID-19 patients, 27.8% exhibited at least one long-term symptom four months post-infection [8]. Similarly, a recent study of 304 non-hospitalized individuals found that 69.1% of them were classified as having post-COVID symptoms [9]. Furthermore, even with longer follow-up periods, a high percentage of non-hospitalized patients experienced persistent symptoms. Kumar S. et al. found that 78.7% of patients had at least one post-COVID-19 symptom at 18-month follow-up [10]. Additionally, among 110 previously hospitalized COVID-19 patients, 74% of them reported at least one ongoing symptom approximately 90 days post-infection [11]. A systematic review and meta-analysis by Fernàndez-de-Las-Penas C. et al. showed that among 15,244 previously hospitalized and 9011 non-hospitalized individuals, 63.2%, 71.9%, and 45.9% exhibited at least one post-COVID symptom at 30 days, 60 days, and more than 90 days post-onset/hospitalization, respectively [12]. Additionally, it has been demonstrated that vaccination against COVID-19 reduces the risk of long-term symptoms [13,14]. Patients who developed the infection after receiving three vaccine doses showed a lower probability of persistent symptoms lasting ≥ 4 weeks, hospitalization, and severe disease compared to those who received only two doses [15].

According to the definition of the National Institute for Health and Care Excellence (NICE), symptoms manifesting after twelve weeks from the infection and lasting for at least two months in individuals with past confirmed or probable SARS-CoV-2 infection and that are not explained by other diagnoses represent the “post-COVID-19 condition” [16]. Furthermore, the tropism of SARS-CoV-2 for the gastrointestinal system can cause an alteration in the intestinal microbiota, leading to immune dysregulation, a prolonged proinflammatory state, mild-to-severe local symptoms, and psychiatric manifestations [17]. In addition, it has been shown that gut-microbiota-related metabolites are impaired in patients with COVID-19, linked with inflammatory cytokines increased during Sars-CoV-2 infection, and connected to symptoms of breathlessness, with lower levels of betaine associated with poor outcomes, likely affecting the immune response [18,19]. Hence, the use of micronutrients and probiotics to reconstitute the physiological microbiome both during the acute phase and in the subsequent weeks aims to decrease the severity of symptoms, such as diarrhea, nausea, vomiting, and abdominal pain and restore exercise capacity. Such nutritional supplementation may contribute to an inhibition of inflammation and an improvement in intestinal homeostasis and the immune system, promoting a reduction in susceptibility to infections [20]. Thus, PIRV-F20^®^, a nutrient supplement constituted by lactoferrin, lysozyme, lactobacillus, resveratrol, vitamins, zinc, and copper—see Table 1—is expected to positively affect post-COVID patients thanks to its demonstrated antiviral, antithrombotic, antioxidant, anti-inflammatory, and immunomodulatory properties. Therefore, the aim of the present retrospective study was to examine what the effect of the administration of PIRV-F20^®^ is on the exercise impairment of patients with post-COVID-19 syndrome. We performed a retrospective observational chart analysis of patients referred to a specific outpatient clinic dedicated to post-COVID-19 syndrome comparing individuals who reported having taken PIRV-F20^®^ to subjects who refused such treatment.

## 2. Materials and Methods

This retrospective and single-center study (Department of Internal Medicine of the University of Naples Federico II) was approved by the Ethics Committee “A.O.U. Federico II—A.O.R.N. Cardarelli” of Naples on 12 April 2023 with Protocol n. 00010689 and was conducted in compliance with the Declaration of Helsinki. All consecutive post-COVID-19 patients of both sexes, aged ≥ 18, with a verified previous SARS-CoV-2 infection in the preceding 12 months—diagnosed by nasopharyngeal swab at least twelve weeks before—any persistent symptom consistent with post-COVID syndrome, and initial exercise impairment, referred to a dedicated outpatient clinic for post-COVID-19 syndrome at the Department of Internal Medicine and Clinical Complexity of the University of Naples Federico II from April 2022 to April 2023, were included in the following analysis, after signing their informed consent. Exercise impairment was defined by a six-minute walking distance during the six-minute walking test ≤ 400 mt. Data of patients were excluded from the analysis if they showed active cancer, end-stage conditions—such as patients awaiting transplantation or severe heart, lung, kidney, or liver dysfunction—any musculoskeletal conditions that prevent the 6 MWT, or patients with pregnancy, breastfeeding, or a history of limited functional capacity. The latter was defined as the incapability to complete a 6 MWT.

### 2.1. Design of the Study and Data Collection

For all these patients, information about symptoms, metabolic profile, physical performance, and cardiac function was collected at the first visit (T0) and after 6 weeks (T1). Examined variables were data related to near and remote medical history, anthropometric data (i.e., weight, height, and body mass index), evaluations regarding muscle strength and exercise capacity through handgrip and 6 MWT plus Borg scale, cardiac function through 12-lead resting electrocardiogram (ECG), and transthoracic echocardiogram at rest (TTE), as well as data from standardized questionnaires about weakness, tiredness, alteration in concentration, dizziness, headache, sleep disorders, and gastrointestinal symptoms. In addition, any side effect in the two groups was recorded. The primary endpoint was a change in 6MWD during the six-minute walking test in the group of patients who reported having taken the treatment when compared to the control group. Secondary endpoints included changes in muscle strength, cardiac function, perception of symptoms, and their impact on quality of life after the therapy with PIRV-F20^®^ among patients who reported having taken the treatment vs. controls.

### 2.2. Study Procedures

In relation to remote pathological history, the physician collected possible previous or current diseases and any allergy to drugs and/or foods or food intolerance. Current medical therapy and data on height and weight were recorded. Furthermore, through standardized questionnaires, information about asthenia, muscle weakness, gastrointestinal symptoms, and their impact on quality of life was gathered. The 6 MWT was performed to evaluate exercise capacity, and during the test, the main measure was the total distance covered in six minutes. Furthermore, oxygen saturation (through a finger pulse oximeter) and the degree of perception of physical exertion and dyspnea during exercise (BORG scale) were also recorded. During the test, the patient walked completely independently back and forth on a 30 m straight pathway free of traffic, and the physician did not walk with the patient or provide him/her support. Data on muscle strength were obtained through the handgrip test, which consists of grasping the dynamometer with each hand and taking three measurements per arm, reporting the highest value as the final measurement. The echocardiographic examination was performed by a single experienced operator (FG) with the Echo Color Doppler GE HealthCare Vivid E95. The electrocardiographic trace was also recorded during image acquisition. Patients were positioned on the left lateral decubitus for parasternal and apical windows and supine for the subcostal view. The imaging views described were the parasternal long-axis (PLAX), parasternal short-axis (PSAX), apical (4-, 2- and 3-chamber), and subcostal. Measurements were obtained using B-mode, M-mode, ColorDoppler (continuous wave—CW, pulsed wave—PW Doppler), and tissue Doppler imaging (TDI) according to the ASE/EACVI recommendations [21,22].

### 2.3. Statistical Analysis

Normally distributed continuous variables were expressed as mean ± standard deviation (SD), whereas continuous data with skewed distributions were expressed as median [interquartile range (IQR)]. Categorical variables were expressed as counts and percentages. The difference between the two groups was investigated through Student’s *t*-test for independent samples or the non-parametric Wilcoxon–Mann–Whitney test when the distribution of the variable under examination was far from normality. Categorical variables were compared between the two groups using the chi-square test. Significance levels <0.05 were accepted. Due to the exploratory nature of this study, the magnitude of the expected result was not predetermined. A sample size calculation was not performed.

## 3. Results

**Figure 1:**
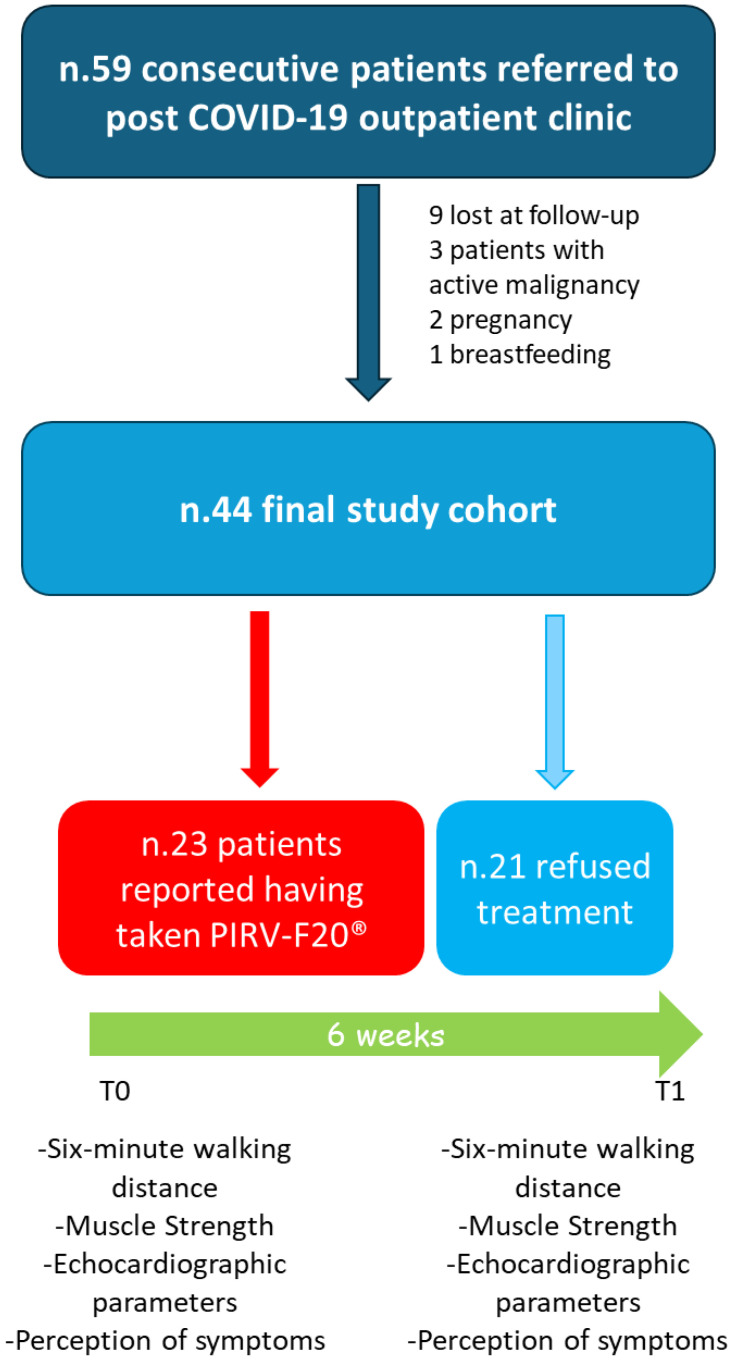
Flow chart of the study, representing the final cohort of patients included and the procedures carried out in the study.

Of the 59 consecutive patients referred to the post-COVID-19 outpatient clinic, 9 were lost at follow-up, 3 had active malignancy, 2 were pregnant, and 1 was breastfeeding. Thus, the final study cohort consisted of 44 patients, including 23 who reported having taken PIRV-F20^®^ compared to 21 who refused this treatment (see Figure 1).

Out of the final cohort accounting for 44 patients, 25 were women (56.8%) and 19 were men (43.2%), aged from 24 to 82 years, with a mean age of 49.1 ± 18.1 years. Most patients included in the current analysis experienced a mild-to-moderate course of acute SARS-CoV-2 infection (cough, dyspnea, and flu-like symptoms as more prevalent complaints) and reported to be treated at home by their general practitioner with symptomatic treatments, mainly NSAID or paracetamol. Of the overall population, only two patients (4.5%) were hospitalized for clinical deterioration and were put on respiratory support for >72 h for lung failure associated with evidence of ground glass bilateral pneumonia. All patients were fully vaccinated according to the current vaccine schedule. Specifically, 21 out of 23 patients (91%) in the group of patients who reported having taken PIRV-F20^®^ were vaccinated with three doses of an mRNA vaccine; precisely 17 of them had the first two doses of Comirnaty and the other four had Spikevax—in both cases followed by the third dose of mRNA vaccine—while 2 subjects (9%) had Vaxzevria for the first two doses followed by a third dose of Comirnaty or Spikevax. The totality of patients in the control group was vaccinated with three doses of Comirnaty. Analyzing separately the two groups, 13 females (56.5%) and 10 males (43.5%) were taken as part of the group of patients who reported having assumed the treatment, and 12 females (57.1%) and 9 males (42.9%) were added to the control group. Among the totality of subjects referred to our outpatient clinic for post-COVID-19 syndrome, only two, in both cases belonging to the group of patients who reported having taken the treatment, described a side effect, represented in one case by cough and in the other by constipation, which promptly disappeared after the discontinuation of therapy. The baseline characteristics of the two groups are compared in Table 2. Regarding anthropometric data (weight, height, and BMI), no statistically significant difference was detected, although there was a significant age difference. Furthermore, no statistically significant difference was observed in relation to serum electrolytes, renal and hepatic function, and metabolic parameters. There was no statistically significant difference in terms of echocardiographic parameters at baseline. In addition, after six weeks of PIRV-F20^®^ no statistically significant differences regarding cardiac function and morphology were observed in the group of patients who reported having taken the treatment in comparison with the controls.

Regarding post-COVID-19 symptoms at baseline, patients reported more impaired concentration, tiredness, weakness, and gastrointestinal symptoms (see Table 3). No statistical differences were reported in the group of patients who reported having taken the six-week therapy with PIRV-F20^®^ when compared with the controls.

**Figure 2:**
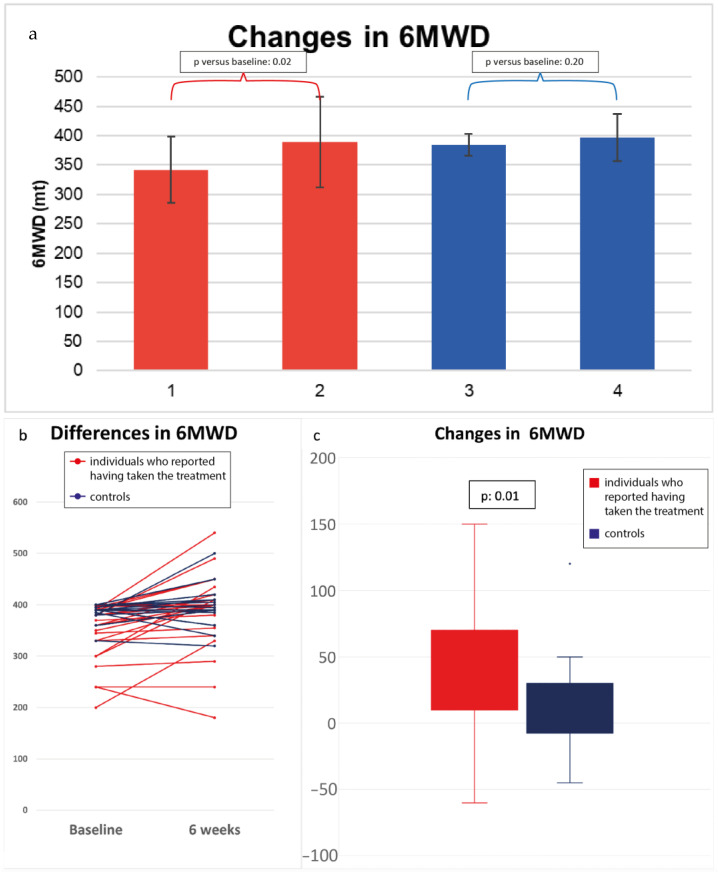
Data on six-minute walking test. (**a**) The panel shows the differences in six-minute walking distance (6MWD) between the two groups at baseline and after 6 weeks, expressed as mean and standard deviation. In red is the group of individuals who reported having taken PIRV-F20^®^, and in blue is the control group. 1 = group of individuals who reported having taken PIRV-F20^®^ at baseline (T0); 2 = group of individuals who reported having taken PIRV-F20^®^ after 6 weeks (T1); 3 = control group at baseline (T0); 4 = control group after 6 weeks (T1). (**b**) In this panel, the figure exhibits for each patient the difference in 6MWD after 6 weeks. (**c**) The panel represents the changes in 6MWD in the group of individuals who reported having taken PIRV-F20^®^ (in red) versus the control group (in blue) after 6 weeks.

The group of individuals who reported having taken PIRV-F20^®^ exhibited a statistically significant difference (*p* = 0.02 vs. 0.20) in terms of 6MWD after the treatment, expressed as mean ± standard deviation (342.1 ± 56.8 at T0 vs. 389.3 ± 77.3 at T1 compared with 384.5 ± 17.9 at T0 vs. 396.9 ± 39.6 for the control group)—see Figure 2—and the difference in the T1-T0 of the 6MWD between the two groups (*p* = 0.01) was represented as median and interquartile range (median: +40 m and IQR: 10–65 m for the group of individuals who reported having taken the treatment vs. median: +10 m and IQR: −5–30 m for the control group). In addition, Figure 2 shows the change in 6MWD for each patient after six weeks and the total changes in 6MWD between the two groups. No difference was shown in dyspnea, measured through the Borg scale pre- and post-treatment. A sensitivity analysis was performed, aimed at overcoming age differences in our study sample. After excluding patients < 35 years in the control group and patients > 70 years in the treatment group, a significance was kept (0.015). A further evaluation in 17 patients after 24 weeks was performed, revealing a further increase of 15.3 ± 2.4 m in the group of people reporting having taken PIRV-F20.

Furthermore, regarding muscle strength, the data did not exhibit statistically significant differences in the two group, as shown in Table 4.

## 4. Discussion

The main findings of the present study are as follows: (i) the post-COVID-19 patients analyzed exhibited more frequent neurologic, muscular, and gastrointestinal symptoms, reporting among these more impaired concentration, tiredness, weakness, and gastrointestinal symptoms; (ii) the use of PIRV-F20^®^ in post-COVID-19 patients with exercise impairment improves the exercise capacity, expressed as the 6MWD. The current study is one of the few evaluating a possible treatment of patients with post-COVID-19 syndrome.

This study was implemented considering the antiviral, antithrombotic, antioxidant, anti-inflammatory, and immunomodulatory properties of this nutrient supplement. Although this must be considered only a pilot study to be carefully interpreted, encouraging results in terms of improving exercise capacity (significant increase in 6MWD change in the group of patients who reported having taken the treatment) after a short period of therapy (i.e., 6 weeks) should be tested in further investigations in the field.

The benefits of the use of this natural supplement are represented by a statistically significant increase in the distance during the test in the group of patients that reported having taken PIRV-F20^®^ for six weeks as compared to patients that did not assume it or did not undergo any other specific therapy for post-COVID-19 syndrome. To the authors’ knowledge, this is the first study on this nutrient supplement, which could be further studied given the present results. The long-term effects of COVID-19 infection can last independently of the severity of the acute disease, and thus far, the underlying pathophysiology and treatment are still not completely defined [23,24]. In fact, in a recent systematic review, the authors identified that the most frequent sequelae of COVID-19 affect the lungs, vessels, heart, and brain [25]. Dysregulation of the renin–angiotensin–aldosterone system due to SARS-CoV-2 develops an increase in angiotensin II, promoting an inflammatory and profibrotic state. This fact leads to a stimulation of cytokine cascade, a rise in interleukin 6 (IL-6) and tumor necrosis factor-α (TNF-α), and subsequently endothelial injury [26]. The release of proinflammatory cytokines leads to an increase in platelet aggregation that results in possible thrombotic occlusion [27]. Beyond the acute phase of this disease, a chronic inflammatory response, due to the persistence of SARS-CoV-2 in the heart, can be involved in myocardial inflammation or ischemia, right ventricular dysfunction, and arrhythmias [28,29]. Furthermore, an immune dysregulation has been shown in patients with long-term COVID-19 symptoms who had mild acute COVID-19 explained in T cell alterations, expressed by a decrease in the number of CD4+ and CD8+ effector memory cells [5,30]. The release of proinflammatory cytokines, i.e., TNFα, IL-6, and IFN-β, and the durable increase in activated monocytes and plasmacytoid dendritic cells, promote a state of persistent inflammation in patients with long-term COVID-19 symptoms [31,32]. One of the possible reasons of our results can be found in lactoferrin, which is one of the main components of PIRV-F20^®^. Indeed, lactoferrin is capable of directly binding at the expense of heparan sulfate proteoglycans (HSPGs) on the host cells’ surface, which are involved in the entry mechanism of coronavirus, in association with a decrease in viral replication due to the activation of intracellular signals [33,34]. Our data cannot claim the capability to demonstrate mechanistic insight into post-COVID-19 syndrome and eventually its treatment, but they may trigger further mechanistic research aimed at investigating the effect of lactoferrin in post-COVID-19 syndrome.

### 4.1. Comparison with Previous Works

In an observational cohort study conducted in the Netherlands, the authors analyzed the nature, prevalence, and severity of long-term symptoms related to COVID-19, evaluating the symptoms presented by the subjects before COVID-19 and 90–150 days after the infection and comparing with matched controls without infection [35]. The study showed that in the COVID-19 population, at least one of the symptoms related to the cardiopulmonary system (i.e., chest pain and dyspnea), musculoskeletal apparatus (i.e., muscles pain), sensory system (i.e., ageusia or anosmia), and general symptoms (i.e., weakness, tiredness) after 90–150 days from COVID-19 was more severe than before the infection or compared to the matched population without COVID-19 [35]. Moreover, in this population, after healing from COVID-19, females depicted a longer persistence of increased symptoms severity in comparison with males [35]. In a study carried out on 247 home-isolated patients [36], in 55% of them, the persistence of COVID-19 symptoms after six months following the infection was registered, mostly represented by fatigue (30%), alteration in taste and/or smell (27%), concentration impairment (19%), memory loss (18%), and dyspnea (15%). In comparison, the cohort of patients analyzed in the present work more frequently exhibited symptoms related to the neurologic, muscular, and gastrointestinal system, with alteration in concentration described as the main symptom (65.9% of subjects), followed by weakness (59.1%), tiredness (54.5%), and gastrointestinal symptoms (50%).

Although there are several studies in the literature on the use of nutrient supplements during acute SARS-CoV-2 infection, such as vitamin D, which showed an inverse correlation between its serum concentration and SARS-CoV-2 positivity and/or COVID-19 incidence, severity, and/or death [37], there is a lack of knowledge on their use in post-COVID-19 syndrome. In fact, very few trials have been conducted to analyze their effects in this category of patients [38], and often, the authors only suggest a possible use of them according to the properties of the support, such as luteolin formulations to treat brain fog associated with long COVID-19 syndrome [39]. In the literature, two clinical cases of pediatric patients were described after presenting to a post-COVID pediatric unit because of persistent gastrointestinal symptoms due to SARS-CoV-2 [40]. In the first case, 16 weeks after the infection, a therapy with high doses (600 mg/die) of oral lactoferrin for 90 days promoted the complete resolution of diarrhea in two weeks and a gradual improvement in fatigue. In addition, in a following SARS-CoV-2 infection, the six-year-old child who was still in treatment with lactoferrin was the only member of the family who did not show any symptoms [40]. In the second case, 4 weeks after SARS-CoV-2 infection, an eleven-month-old baby with persistent diarrhea was treated with oral lactoferrin 400 mg/die for 90 days, exhibiting an improvement in gastrointestinal symptoms already in one week [40].

### 4.2. Clinical Implications

Since the antiviral, antithrombotic, antioxidant, anti-inflammatory, and immunomodulatory properties of this nutrient supplement show benefits in the improvement in exercise capacity in post-COVID-19 patients, the use of PIRV-F20^®^ might be investigated in other diseases that are characterized by inflammation, oxidation, and dysregulation in the immune system. This therapy, administered during the acute phase of SARS-CoV-2 infection, might show efficacy in decreased persistence or onset of symptoms after recovery. Further studies might investigate the possible benefits of PIRV-F20^®^ in reducing susceptibility to viral infections.

### 4.3. Limitations of the Study

The main limitation of the current work is its retrospective nature. According to the nature of the study, there was not a study group assuming a placebo, which has meant that we could not evaluate the possible “placebo effect”. Although the population studied represents only a limited group, due to the small number of patients evaluated, the results are encouraging. Furthermore, the difference in age of the subjects in the group of patients who reported having taken the treatment in comparison with those in the control group depicts a further limitation of the current study. However, despite the older age, patients who reported having taken PIRV-F20^®^ showed a greater improvement in exercise capacity when compared with the controls. The control group was composed of subjects refusing treatment mainly because of skepticism about its effectiveness and, especially for workers and for those who did not take chronic therapy, the difficulty in complying with a daily therapy. Although we acknowledge this as a potential selection bias, the two samples were almost comparable. Furthermore, data on the patients’ compliance with the treatment were not systematically assessed. However, although self-reported, all patients reported a treatment adherence > 95%. As another limitation of the study, given its exploratory nature, the magnitude of the expected result was not predetermined. Also, a sample size calculation was not performed. Therefore, our results should be interpreted cautiously.

## 5. Conclusions

The findings of the present study suggest that PIRV-F20^®^ might possibly improve exercise capacity in patients affected by post-COVID-19 syndrome through the increase in six-minute walking distance during the six-minute walking test, despite the older age of the patients who reported having taken the treatment in comparison with the controls. Secondary endpoints such as changes in muscle strength, echocardiographic parameters, and perception of symptoms related to post-COVID-19 condition did not show significant differences. Therefore, our results, although promising, should be carefully interpreted, possibly in prospective randomized controlled clinical trials.

## Figures and Tables

**Table 1:** Main components of the nutrient supplement PIRV-F20^®^. NRVs: nutrient reference values.

Components of PIRV-F20^®^	Mean Values per Dose
*Lactobacillus paracasei* subsp. paracasei F19	6 × 10^9^
Lysozyme	250 mg
Lactoferrin	30 mg
Resveratrol	20 mg
A Vitamin	1.200 mcg (150% NRV)
C Vitamin	30 mg (37.5% NRV)
D3 Vitamin	25 mcg (1.000 UI) (500% NRV)
E Vitamin	40 mg (333.3% NRV)
K2 Vitamin	11.25 mcg (15% NRV)
Zinc	7.5 mg (75% NRV)
Copper	2 mg (200% NRV)

**Table 2:** Characteristics at baseline of the study population.

	Overall (44 pts)	Patients Who Reported Having Taken PIRV-F20^®^ (23 pts)	Controls (21 pts)	*p*
Anthropometric characteristics				
Female (%)	25 (56.8%)	13 (56.5%)	12 (57.1%)	
Age (years) ± SD	49.1 ± 18.1	57.8 ± 15.2	39.7 ± 16.4	<0.001
Weight (kg) ± SD	77.6 ± 22.6	81.2 ± 25.8	73.7 ± 18.3	0.28
Height (cm) ± SD	167.4 ± 8.3	166.9 ± 9.5	168.0 ± 7.0	0.66
BMI (Kg/m^2^) ± SD	27.5 ± 6.7	28.8 ± 6.7	26.1 ± 6.5	0.18
Blood test				
Na^+^ (mmol/L) ± SD	140.7 ± 2.0	140.7 ± 1.7	140.7 ± 2.2	0.97
K^+^ (mmol/L) ± SD	4.2 ± 0.4	4.3 ± 0.4	4.1 ± 0.3	0.17
Ca^2+^ (mg/dl) ± SD	9.2 ± 0.5	9.1 ± 0.5	9.3 ± 0.5	0.12
Glycemia (mg/dL) ± SD	87.6 ± 15.3	86.8 ± 15.1	88.6 ± 15.9	0.69
Creatinine (mg/dL) ± SD	0.9 ± 0.1	0.9 ± 0.1	0.8 ± 0.1	0.14
Urea (mg/dl) ± SD	40.0 ± 10.0	42.3 ± 11.5	37.4 ± 7.6	0.10
AST (U/L) ± SD	24.4 ± 13.4	25.6 ± 17.9	23.1 ± 5.7	0.54
ALT (U/L) ± SD	25.7 ± 14.7	24.7 ± 18.0	26.8 ± 10.5	0.65
Total cholesterol (mg/dL) ± SD	170.8 ± 30.5	170. 4 ± 39.8	171.3 ± 16.1	0.93
LDL cholesterol (mg/dL) ± SD	91.4 ± 30.8	98.1 ± 40.3	84.1 ± 12.2	0.13
Triglycerides (mg/dL) ± SD	98.8 ± 32.8	102.9 ± 44.1	94.3 ± 11.7	0.39
C-reactive protein (mg/L) ± SD	0.6 ± 0.7	0.7 ± 0.9	0.6 ± 0.5	0.72
Vitamin D (ng/mL) ± SD	23.1 ± 7.4	21.2 ± 8.4	25.0 ± 5.7	0.09
Vital signs				
SBP (mmHg) ± SD	126.5 ± 13.3	130.0 ± 14.1	122.6 ± 11.5	0.06
DBP (mmHg) ± SD	78.1 ± 8.2	79.1 ± 10.0	77.0 ± 5.9	0.40
O_2_ saturation (%) ± SD	97.3 ± 2.2	96.9 ± 2.2	97.8 ± 2.1	0.13
Heart rate (bpm) ± SD	77.1 ± 14.0	77.0 ± 14.3	77.1 ± 14.0	0.97
Comorbidities				
Hypertension	18 (41.0%)	12 (52.2%)	6 (28.6%)	0.11
Obesity	11 (25.0%)	7 (30.4%)	4 (19.0%)	0.38
Hypercholesterolemia	18 (41.0%)	12 (52.2%)	6 (28.6%)	0.11
CAD	2 (4.5%)	2 (8.7%)	0	0.17
Diabetes mellitus	4 (9.1%)	3 (13.0%)	1 (4.8%)	0.34
CKD (eGFR < 60 mL/min/1.73)	3 (6.8%)	3 (13.0%)	0	0.09
Pulmonary embolism	1 (2.3%)	1 (4.3%)	0	0.33
Chronic respiratory failure	3 (6.8%)	2 (8.7%)	1 (4.8%)	0.60
Allergic asthma	3 (6.8%)	3 (13.0%)	0	0.09
Pulmonary fibrosis	4 (9.1%)	3 (13.0%)	1 (4.8%)	0.34
OSAS	3 (6.8%)	3 (13.0%)	0	0.09
COPD	3 (6.8%)	2 (8.7%)	1 (4.8%)	0.60
Hospitalization due to COVID-19	2 (4.5%)	1 (4.3%)	1 (4.8%)	0.95
Echocardiographic parameters				
TRV (m/s)	2.1 ± 0.3	2.1 ± 0.4	2.0 ± 0.3	0.20
RAA (cm^2^)	14.4 ± 2.3	14.3 ± 2.5	14.6 ± 2.0	0.65
TAPSE (mm)	23.8 ± 2.2	23.4 ± 2.7	24.2 ± 1.6	0.22
LVEF (%)	59.5 ± 3.7	59.0 ± 4.4	60.0 ± 2.8	0.35
IVC (mm)	11.4 ± 2.9	10.7 ±2.7	12.0 ± 3.1	0.14

Legend: ALT, alanine transaminase; AST, aspartate transaminase; BMI, body mass index; Ca^2+^, calcium; CAD, coronary artery disease; CKD, chronic kidney disease; COPD, chronic obstructive pulmonary disease; DBP, diastolic blood pressure; eGFR, estimated glomerular filtration rate; IVC, inferior vena cava; K^+^, potassium; LVEF, left ventricular ejection fraction; Na^+^, sodium; O_2_, oxygen; OSAS, obstructive sleep apnea syndrome; RAA, right atrium area; SBP, systolic blood pressure; SD, standard deviation; TAPSE, tricuspid annular plane systolic excursion; TRV, tricuspid regurgitation velocity.

**Table 3:** Main post-COVID-19 symptoms complained by patients at baseline.

	Overall (44 pts)	Patients Who Reported Having Taken PIRV-F20^®^ (23 pts)	Controls (21 pts)	*p*
Weakness	26 (59.1%)	16 (69.6%)	10 (47.6%)	0.14
Tiredness	24 (54.5%)	14 (60.9%)	10 (47.6%)	0.38
Alteration in concentration	29 (65.9%)	17 (73.9%)	12 (57.1%)	0.24
Dizziness	10 (22.7%)	7 (30.4%)	3 (14.3%)	0.20
Headache	18 (40.9%)	12 (52.2%)	6 (28.6%)	0.11
Sleep disorders	14 (31.8%)	10 (43.5%)	4 (19.0%)	0.08
Cognitive impairment	3 (6.8%)	2 (8.7%)	1 (4.8%)	0.60
Gastrointestinal symptoms	22 (50.0%)	12 (52.2%)	10 (47.6%)	0.76

**Table 4:** Data on handgrip. Changes in each group in terms of muscle strength of the right and left hand comparing the measurements at baseline (T0) with those at 6 weeks (T1).

Handgrip						
	Patients Who Reported Having Taken PIRV-F20^®^			Controls		
	T0	T1	*p*	T0	T1	*p*
Right hand (Kg) [mean ± SD]	22.7 ± 12.2	24.1 ± 17.9	0.78	27.6 ± 10.8	28.1 ± 9.7	0.89
Left hand (Kg) [mean ± SD]	20.1 ± 8.5	22.0 ± 12.2	0.58	24.6 ± 7.8	26.4 ± 9.8	0.51
ΔRight hand T1−T0 (median [IQR])	0 [−3–2.5]			2 [−1.75–2]		0.7
ΔLeft hand T1−T0 (median [IQR])	0.5 [−2.25–4]			2 [−0.5–5.25]		0.5

Legend: ΔLeft hand T1-T0, difference in grip strength of the left hand between 6-week (T1) and baseline (T0) measurements, expressed as median and interquartile range; ΔRight hand T1-T0, difference in grip strength of the right hand between 6-week (T1) and baseline (T0) measurements, expressed as median and interquartile range; SD, standard deviation.

## Data Availability

The data presented in this study are available on request from the corresponding author due to patients’ privacy reasons.

## References

[1] Wang M.Y., Zhao R., Gao L.J., Gao X.F., Wang D.P., Cao J.M. (2020). SARS-CoV-2: Structure, Biology, and Structure-Based Therapeutics Development. Front. Cell. Infect. Microbiol..

[2] Wiersinga W.J., Rhodes A., Cheng A.C., Peacock S.J., Prescott H.C. (2020). Pathophysiology, Transmission, Diagnosis, and Treatment of Coronavirus Disease 2019 (COVID-19): A Review. JAMA.

[3] Jackson C.B., Farzan M., Chen B., Choe H. (2022). Mechanisms of SARS-CoV-2 entry into cells. Nat. Rev. Mol Cell Biol..

[4] Wang Y., Wang Y., Luo W., Huang L., Xiao J., Li F., Qin S., Song X., Wu Y., Zeng Q. (2020). A comprehensive investigation of the mRNA and protein level of ACE2, the putative receptor of SARS-CoV-2, in human tissues and blood cells. Int. J. Med. Sci..

[5] Davis H.E., McCorkell L., Vogel J.M., Topol E.J. (2023). Long COVID: Major findings, mechanisms and recommendations. Nat. Rev. Microbiol..

[6] Ceban F., Ling S., Lui L.M.W., Lee Y., Gill H., Teopiz K.M., Rodrigues N.B., Subramaniapillai M., Di Vincenzo J.D., Cao B. (2022). Fatigue and cognitive impairment in Post-COVID-19 Syndrome: A systematic review and meta-analysis. Brain Behav. Immun..

[7] Lopez-Leon S., Wegman-Ostrosky T., Perelman C., Sepulveda R., Rebolledo P.A., Cuapio A., Villapol S. (2021). More than 50 long-term effects of COVID-19: A systematic review and meta-analysis. Sci Rep..

[8] Augustin M., Schommers P., Stecher M., Dewald F., Gieselmann L., Gruell H., Horn C., Vanshylla K., Cristanziano V.D., Osebold L. (2021). Post-COVID syndrome in non-hospitalised patients with COVID-19: A longitudinal prospective cohort study. Lancet Reg. Health Eur..

[9] Kirchberger I., Meisinger C., Warm T.D., Hyhlik-Dürr A., Linseisen J., Goßlau Y. (2023). Post-COVID-19 Syndrome in Non-Hospitalized Individuals: Healthcare Situation 2 Years after SARS-CoV-2 Infection. Viruses.

[10] Kumar S., Patidar V., Mudgal S.K., Kumar S., Agarwal R., Gupta P., Gaur R., Varshney S. (2023). Self-Reported Persistent Symptoms at 18 Months and Above Among COVID-19 Non-hospitalized Patients: A Prospective Cohort Study. Cureus.

[11] Arnold D.T., Hamilton F.W., Milne A., Morley A.J., Viner J., Attwood M., Noel A., Gunning S., Hatrick J., Hamilton S. (2021). Patient outcomes after hospitalisation with COVID-19 and implications for follow-up: Results from a prospective UK cohort. Thorax.

[12] Fernández-de-Las-Peñas C., Palacios-Ceña D., Gómez-Mayordomo V., Florencio L.L., Cuadrado M.L., Plaza-Manzano G., Navarro-Santana M. (2021). Prevalence of post-COVID-19 symptoms in hospitalized and non-hospitalized COVID-19 survivors: A systematic review and meta-analysis. Eur. J. Intern. Med..

[13] Català M., Mercadé-Besora N., Kolde R., Trinh N.T.H., Roel E., Burn E., Rathod-Mistry T., Kostka K., Man W.Y., Delmestri A. (2024). The effectiveness of COVID-19 vaccines to prevent long COVID symptoms: Staggered cohort study of data from the UK, Spain, and Estonia. Lancet Respir. Med..

[14] Al-Aly Z., Bowe B., Xie Y. (2022). Long COVID after breakthrough SARS-CoV-2 infection. Nat. Med..

[15] Antonelli M., Penfold R.S., Canas L.D.S., Sudre C., Rjoob K., Murray B., Molteni E., Kerfoot E., Cheetham N., Pujol J.C. (2023). SARS-CoV-2 infection following booster vaccination: Illness and symptom profile in a prospective, observational community-based case-control study. J. Infect..

[16] Soriano J.B., Murthy S., Marshall J.C., Relan P., Diaz J.V., WHO Clinical Case Definition Working Group on Post-COVID-19 Condition (2022). A clinical case definition of post-COVID-19 condition by a Delphi consensus. Lancet Infect. Dis..

[17] Yeoh Y.K., Zuo T., Lui G.C., Zhang F., Liu Q., Li A.Y., Chung A.C., Cheung C.P., Tso E.Y., Fung K.S. (2021). Gut microbiota composition reflects disease severity and dysfunctional immune responses in patients with COVID-19. Gut.

[18] Israr M.Z., Ibrahim W., Salzano A., Sarmad S., Wilde M.J., Cordell R.L., Greening N.J., Brightling C.E., Siddiqui S., Suzuki T. (2022). Association of gut-related metabolites with respiratory symptoms in COVID-19: A proof-of-concept study. Nutrition.

[19] Nagata N., Takeuchi T., Masuoka H., Aoki R., Ishikane M., Iwamoto N., Sugiyama M., Suda W., Nakanishi Y., Terada-Hirashima J. (2023). Human Gut Microbiota and Its Metabolites Impact Immune Responses in COVID-19 and Its Complications. Gastroenterology.

[20] Dhar D., Mohanty A. (2020). Gut microbiota and COVID-19- possible link and implications. Virus Res..

[21] Mitchell C., Rahko P.S., Blauwet L.A., Canaday B., Finstuen J.A., Foster M.C., Horton K., Ogunyankin K.O., Palma R.A., Velazquez E.J. (2019). Guidelines for Performing a Comprehensive Transthoracic Echocardiographic Examination in Adults: Recommendations from the American Society of Echocardiography. J. Am. Soc. Echocardiogr..

[22] Galderisi M., Cosyns B., Edvardsen T., Cardim N., Delgado V., Di Salvo G., Donal E., Sade L.E., Ernande L., Garbi M. (2017). Standardization of adult transthoracic echocardiography reporting in agreement with recent chamber quantification, diastolic function, and heart valve disease recommendations: An expert consensus document of the European Association of Cardiovascular Imaging. Eur. Heart J. Cardiovasc. Imaging.

[23] Desai A.D., Lavelle M., Boursiquot B.C., Wan E.Y. (2022). Long-term complications of COVID-19. Am. J. Physiol. Cell Physiol..

[24] Jin Y., Ji W., Yang H., Chen S., Zhang W., Duan G. (2020). Endothelial activation and dysfunction in COVID-19: From basic mechanisms to potential therapeutic approaches. Signal Transduct. Target Ther..

[25] SeyedAlinaghi S., Afsahi A.M., MohsseniPour M., Behnezhad F., Salehi M.A., Barzegary A., Mirzapour P., Mehraeen E., Dadras O. (2021). Late Complications of COVID-19; a Systematic Review of Current Evidence. Arch. Acad. Emerg. Med..

[26] McDonald L.T. (2021). Healing after COVID-19: Are survivors at risk for pulmonary fibrosis?. Am. J. Physiol. Lung Cell Mol. Physiol..

[27] Wang F., Kream R.M., Stefano G.B. (2020). Long-Term Respiratory and Neurological Sequelae of COVID-19. Med. Sci. Monit..

[28] Kang Y., Chen T., Mui D., Ferrari V., Jagasia D., Scherrer-Crosbie M., Chen Y., Han Y. (2020). Cardiovascular manifestations and treatment considerations in COVID-19. Heart.

[29] Raman B., Bluemke D.A., Lüscher T.F., Neubauer S. (2022). Long COVID: Post-acute sequelae of COVID-19 with a cardiovascular focus. Eur. Heart J..

[30] Glynne P., Tahmasebi N., Gant V., Gupta R. (2022). Long COVID following mild SARS-CoV-2 infection: Characteristic T cell alterations and response to antihistamines. J. Investig. Med..

[31] Mehandru S., Merad M. (2022). Pathological sequelae of long-haul COVID. Nat. Immunol..

[32] Low R.N., Low R.J., Akrami A. (2023). A review of cytokine-based pathophysiology of Long COVID symptoms. Front. Med..

[33] Cerezo-Magaña M., Bång-Rudenstam A., Belting M. (2023). Proteoglycans: A common portal for SARS-CoV-2 and extracellular vesicle uptake. Am. J. Physiol. Cell Physiol..

[34] Chang R., Ng T.B., Sun W.Z. (2020). Lactoferrin as potential preventative and adjunct treatment for COVID-19. Int. J. Antimicrob. Agents.

[35] Ballering A.V., van Zon S.K.R., Olde Hartman T.C., Rosmalen J.G.M., Lifelines Corona Research Initiative (2022). Persistence of somatic symptoms after COVID-19 in the Netherlands: An observational cohort study. Lancet.

[36] Blomberg B., Mohn K.G., Brokstad K.A., Zhou F., Linchausen D.W., Hansen B.A., Lartey S., Onyango T.B., Kuwelker K., Sævik M. (2021). Long COVID in a prospective cohort of home-isolated patients. Nat. Med..

[37] Mercola J., Grant W.B., Wagner C.L. (2020). Evidence Regarding Vitamin D and Risk of COVID-19 and Its Severity. Nutrients.

[38] Bradbury J., Wilkinson S., Schloss J. (2023). Nutritional Support During Long COVID: A Systematic Scoping Review. J. Integr. Complement. Med..

[39] Theoharides T.C., Cholevas C., Polyzoidis K., Politis A. (2021). Long-COVID syndrome-associated brain fog and chemofog: Luteolin to the rescue. Biofactors.

[40] Morello R., De Rose C., Cardinali S., Valentini P., Buonsenso D. (2022). Lactoferrin as Possible Treatment for Chronic Gastrointestinal Symptoms in Children with Long COVID: Case Series and Literature Review. Children.

